# Identification of calnexin as a diacylglycerol acyltransferase-2 interacting protein

**DOI:** 10.1371/journal.pone.0210396

**Published:** 2019-01-07

**Authors:** Curtis Brandt, Pamela J. McFie, Huyen Vu, Paulos Chumala, George S. Katselis, Scot J. Stone

**Affiliations:** 1 Department of Biochemistry, Microbiology and Immunology, University of Saskatchewan, Saskatoon, Saskatchewan, Canada; 2 Department of Medicine and the Canadian Centre for Health and Safety in Agriculture, University of Saskatchewan, Saskatoon, Saskatchewan, Canada; University of Alberta, CANADA

## Abstract

Triacylglycerol synthesis is catalyzed by acyl CoA:diacylglycerol acyltransferase-2 (DGAT2). DGAT2 is an integral membrane protein that is localized to the endoplasmic reticulum and interacts with lipid droplets. Using BioId, a method to detect proximal and interacting proteins, we identified calnexin as a DGAT2-interacting protein. Co-immunoprecipitation and proximity ligation assays confirmed this finding. We found that calnexin-deficient mouse embryonic fibroblasts had reduced intracellular triacylglycerol levels and fewer large lipid droplets (>1.0 μm^2^ area). Despite the alterations in triacylglycerol metabolism, *in vitro* DGAT2 activity, localization and protein stability were not affected by the absence of calnexin.

## Introduction

Diacylglycerol acyltransferase (DGAT)-2 is an integral membrane protein that resides in the endoplasmic reticulum (ER) where it catalyzes the final step of triacylglycerol (TG) biosynthesis [1, 2]. DGAT2 has two transmembrane domains separated by a short loop (~5–8 amino acids) that extends into the ER lumen [3, 4]. The first transmembrane domain contains an ER targeting signal while the bulk of DGAT2 C-terminal to its second transmembrane is exposed to the cytosol [4, 5].

DGAT2 catalyzes the formation of TG using fatty acyl coenzyme A (CoA) and 1,2-diacylglycerol (DG) as substrates. TG is the major form of stored metabolic energy in eukaryotic organisms that is sequestered in the hydrophobic core of cytosolic lipid droplets until it is needed. Although present in the ER, DGAT2 also co-localizes with lipid droplets where it is believed to catalyze localized TG synthesis for lipid droplet growth [2].

Chemical cross-linking experiments have demonstrated that DGAT2 is part of a high molecular weight (~650 kD) complex [6]. In earlier studies, a protein complex was partially purified from rat intestine that had DGAT, acyl CoA synthetase, acyl CoA hydrolase and MGAT activities [7]. A similar protein complex was identified in oleaginous yeast [8]. In more current studies, stearoyl CoA desaturase-1, fatty acid transporter-1 and monoacylglycerol acyltransferase-2 have been shown to interact with DGAT2, presumably channeling lipid substrates for efficient TG synthesis [6, 9, 10]. The purpose of this study was to identify additional proteins that interact with DGAT2. Using a biotin ligase/DGAT2 fusion protein, we found that calnexin, an ER chaperone, interacts with DGAT2.

## Methods

### Antibodies

Rabbit anti-calnexin (Enzo Life Sciences: ADI-SPA-865), rabbit anti-DGAT2 (polyclonal antibody generated against the C-terminus of DGAT2 [4], mouse anti-PDI (Abcam: ab2792), mouse anti-Myc (clone 9E10), goat anti-mouse IgG conjugated to Alexa Fluor 594 (Thermo Fisher: A11005), mouse anti-FLAG (Sigma: F3165), goat anti-rabbit IgG conjugated to horseradish peroxidase (Bio-Rad Laboratories: 170–6515), goat anti-mouse IgG conjugated to horseradish peroxidase (Bio-Rad Laboratories: 170–6516), rabbit anti-GAPDH (Stressgen), streptavidin-Alexa Fluor 488 (Thermo Fisher: S11223), streptavidin-HRP (Thermo Fisher: N100), mouse anti-HSP70 (heat shock protein 70; Thermo Fisher: MA3-028).

### Cell culture

COS-7, HEK-293T and mouse 3T3-L1 pre-adipocytes (American Type Culture Collection) were maintained in Dulbecco’s modified Eagle’s medium (DMEM) with 10% fetal bovine serum in a 37 °C incubator with 5% CO_2_. Wild-type (*Cnx2*^*+/+*^) and calnexin-deficient (*Cnx2*^*–/–*^) mouse embryonic fibroblasts (MEFs), were a gift from Dr. M. Michalak (University of Alberta), and were maintained as described above [11].

### Expression and detection of BioId/DGAT2

Murine DGAT2 was fused, in frame, to the C-terminus of Myc-BirA (BioId) [12], a gift from Kyle Roux (Addgene plasmid #35700). HEK-293T cells were transfected with BioId/DGAT2, in pcDNA3.1, using 0.1% polyethylenimine [4]. Biotinylation was stimulated by adding 50 μM biotin to the cell culture media for 12 h. Cells expressing BioId/DGAT2 were lysed by passing the cell suspension through a 27-gauge needle 20 times. Cell debris was pelleted by centrifugation at 1,000 x g and the cell lysate was transferred to a new tube. Equal volumes of cell lysate and 2X Laemmli buffer with 5% β-mercaptoethanol were mixed and boiled for 5 min. Protein samples were separated by SDS-PAGE (10%) and then transferred to a PVDF membrane. BioId/DGAT2 and biotinylated proteins were detected with mouse anti-Myc (1:10 dilution) and streptavidin-HRP (1:10,000 dilution), respectively.

### Fluorescence microscopy

Cells cultured on glass coverslips were fixed with 4% paraformaldehyde in phosphate-buffered saline (PBS) for 10 min followed by permeabilization of cellular membranes with 0.2% Triton X-100 in PBS for 5 min at room temperature. Lipid droplets were detected by staining cells with Bodipy 493/503 (Molecular Probes). Coverslips were mounted on glass slides with mounting medium containing 4',6-diamidino-2-phenylindole (DAPI) to visualize nuclei. Images were obtained using a Zeiss LSM700 laser scanning confocal microscope. Image analyses were done using Fiji [13]. For BioId experiments, cells were incubated with mouse anti-Myc (1:50 dilution)/goat anti-mouse-594 (1:200 dilution) and streptavidin-488 (1:50 dilution) to visualize the BioId/DGAT2 fusion protein and biotinylated proteins, respectively.

### Affinity purification of biotinylated proteins

Cells were lysed in 600 μL of 50 mM Tris-Cl (pH 7.4) containing 500 mM NaCl and 0.2% SDS. Sixty microliters of 20% Triton X-100 was then added followed by probe sonication. Samples were diluted by the addition of 4.32 mL of 50 mM Tris-Cl (pH 7.4) and re-sonicated. Insoluble material was removed by centrifugation at 16,500 x g for 10 min and the solubilized material was transferred to a fresh tube containing 50 μL magnetic streptavidin beads equilibrated in 50 mM Tris-Cl (pH 7.4) containing 250 mM NaCl and 0.1% SDS which was incubated overnight at 4 °C. The magnetic beads were first washed with 1.5 mL 2% SDS, followed by 1% Triton X-100, 1 mM EDTA, 0.1% deoxycholate, 500 mM NaCl in 50 mM Hepes (pH 7.5), and then with 0.5% deoxycholate, 0.5% Nonidet-P40, 1 mM EDTA 250 mM LiCl in 10 mM Tris-Cl (pH 7.4). Beads were collected between each wash using a magnetic stand (Thermo Fisher). After washing, the beads were resuspended in 1.5 mL 50 mM Tris-Cl (pH 7.4).

To elute biotinylated proteins, the beads were pelleted and resuspended in 50 μL of 50 mM ammonium bicarbonate (NH_4_HCO_3_). Beads were isolated using a magnetic stand and the eluted proteins were transferred to a new tube. This step was repeated with 30 μL of 50 mM NH_4_HCO_3_. An aliquot was analyzed by immunoblotting with streptavidin-HRP. Eluted proteins were separated by SDS-PAGE and stained with Bio-Safe Coomassie Brilliant Blue G-250 stain (Bio-Rad). Protein bands of interest were excised from the gel and proteins were identified by mass spectrometry.

### Protein identification by mass spectrometry

Gel slices were destained twice with 100 μL of 200 mM NH_4_HCO_3_ in 50% acetonitrile at 30 °C for 20 min. Gel samples were then treated with acetonitrile for 10 min and dried with a speed-vac. Proteins were reduced with 100 μL of 10 mM dithiothreitol in 100 mM NH_4_HCO_3_ and incubated at 56 °C for 1 hour. Dithiothreitol was removed and replaced with 100 μL 100 mM iodoacetamide and incubated at room temperature in the dark for 30 min. After washing twice with 200 mM NH_4_HCO_3_, samples were shrunk with acetonitrile, re-swelled with 200 mM NH_4_HCO_3_ and re-shrunk with acetonitrile. Samples were dried with a speed-vac and sequentially re-swelled in 20 μL trypsin buffer (50 ng/μL trypsin in 1 mM hydrochloric acid and 100 mM NH_4_HCO_3_) and 30 μL of 200 mM NH_4_HCO_3_. Proteins were digested overnight at 30 °C with shaking (300 rpm). Trypsin action was quenched with 1% trifluoroacetic acid and tryptic peptides were extracted from gel slices in 100 μL of 0.1% trifluoroacetic acid in 60% acetonitrile. Extracted tryptic peptides were dried with a speed-vac and stored at -80 °C until analyzed by mass spectrometry.

### Mass Spectrometry (MS) Workflow

Tryptic peptide were reconstituted in 12 μL of MS grade water:acetonitrile:formic acid (97:3:0.1 v/v). Insoluble material was removed by centrifugation at 18,000 x g for 10 min at 4 °C. A 10 μL aliquot was used for liquid chromatography-tandem mass spectrometry (LC-MS/MS) analysis. Mass spectral analyses were performed on an Agilent 6550 iFunnel quadrupole time-of-flight (QTOF) mass spectrometer equipped with an Agilent 1260 series liquid chromatography instrument and an Agilent Chip Cube LC-MS interface (Agilent Technologies Canada). Chromatographic peptide separation was accomplished using a high-capacity Agilent HPLC-Chip II: G4240-62030 Polaris-HR-Chip-3C18 consisting of a 360 nL enrichment column and a 75 μm × 150 mm analytical column, both packed with Polaris C18-A, 180Å, 3 μm stationary phase. Samples were loaded onto the enrichment column with 0.1% formic acid in water at a flow rate of 2.0 μL min^-1^. After loading onto the analytical column, peptides were separated with a linear gradient solvent system consisting of solvent A (0.1% formic acid in water) and solvent B (0.1% formic acid in acetonitrile). The linear gradient was 3–25% solvent B for 50 min and then 25–90% solvent B for 10 min at a flow rate of 0.3 μL min^-1^. Positive-ion electrospray mass spectral data were acquired using a capillary voltage set at 1900 V, the ion fragmentor set at 360 V, and the drying gas (nitrogen) set at 225 °C with a flow rate of 12.0 L min^-1^. Spectral results were collected over a mass range of 250–1700 (mass/charge; m/z) at a scan rate of 8 spectra s^-1^. MS/MS data were collected over a range of 50–1700 m/z and a set isolation width of 1.3 atomic mass units. A maximum of 20 precursor ions were selected for auto MS/MS at an absolute threshold of 3000 counts and a relative threshold of 0.01% with a 0.25 min active exclusion.

### Peptide Identification

MS/MS spectral data were extracted from raw data and processed against the concatenated SwissProt Human database (UniProt release 2016_06), using Spectrum Mill (Agilent Technologies Canada Ltd., Mississauga, ON, CA) as the database search engine. Search parameters included a fragment mass error of 50 ppm, a parent mass error of 20 ppm, trypsin cleavage specificity (two missed cleavages per peptide), and carbamidomethylation as a fixed modification of cysteine. Oxidized methionine, carbamylated lysine, pyroglutamic acid, deamidated asparagine, phosphorylated serine, threonine, and tyrosine and acetyl lysine were set as variable modifications. Data were also searched using semi-trypsin non-specific C- and N-terminus to increase protein identification. SpectrumMill validation was performed at peptide and protein levels (1% false discovery rate, FDR). Background proteins that included keratins, histones, and ribosomal proteins were removed and not analyzed further. Proteins identified from parental HEK-293T cells were used to minimize false positive candidates [12, 14].

### Co-immunoprecipitation

HEK-293T cells were transfected with plasmids containing FLAG-DGAT2 (FL-DGAT2) or Myc-DGAT2. Twenty hours post-transfection, cellular material was solubilized with 0.5% CHAPS detergent in PBS (detergent:protein ratio of 10:1). Insoluble material was removed by centrifugation and the solubilized material was transferred to a fresh tube and diluted to 2 mL. An aliquot (2.5% of the total) was removed to determine protein abundance (input). Anti-FLAG agarose beads were added to the samples which were rotated for 3 h. Beads were washed with 0.5% CHAPS in PBS and bound proteins were eluted with 70 μL 0.5% CHAPS in PBS containing 150 ng/μL FLAG peptide. Thirty microliter aliquots of the immunoprecipitates were analyzed by immunoblotting with anti-DGAT2, anti-calnexin and anti-PDI antibodies. All experiments were performed at 4 °C.

### Proximity ligation assay (PLA)

COS-7 cells expressing either FL-DGAT2 or Myc-DGAT2 were fixed and permeabilized as described previously. To minimize non-specific binding of antibodies, cells were incubated with 3% BSA in PBS, followed by rabbit anti-calnexin (1:200 dilution) and mouse anti-FLAG (1:200 dilution) or anti-Myc antibodies (1:200 dilution) for 30 min. at 37 °C. The *in situ* proximity ligation assay was performed according to manufacturer’s instructions (Olink Bioscience). Cells were mounted on glass slides in mounting medium with DAPI to visualize nuclei. Images were obtained and analyzed as described for fluorescence microscopy.

### Adipocyte differentiation

3T3-L1 pre-adipocytes were grown to confluence. Cells were then treated with 5 μg/mL insulin, 500 μM 3-Isobutyl-1-methylxanthine, 1 μM dexamethasone and 10 μM troglitazone in DMEM containing 10% FBS. After 24 h, cells were fed fresh differentiation media. At 96 h, cells were incubated with 5 μg/mL insulin for 48 h followed by DMEM and 10% FBS with no supplements.

### *In vitro* DGAT assay

DGAT activity was determined by measuring the formation of N-[(7-nitro-2-1,3-benzoxadiazol-4-yl)-methyl]amino (NBD)-TG from NBD-palmitoyl-CoA [15]. The assay reaction mixture consisted of 100 mM Tris-HCl (pH 7.6), 20 mM MgCl2, 0.625 mg/mL of BSA (fatty acid free), 200 μM 1,2 dioleoyl-sn-glycerol, 25 μM NBD-palmitoyl CoA (Avanti Polar Lipids), 100 mM sucrose and 50 μg of protein sample in a final volume of 200 μL which was incubated at 37 °C for 10 min. The reaction was stopped with the addition of 4 mL chloroform/methanol (2:1, v/v) and 800 μL of water. Reaction products in the organic phase were extracted and separated by thin layer chromatography using the solvent system diethyl ether/hexane/methanol/acetic acid (55:45:5:1, v/v/v/v). NBD-triacylglycerol was detected with a VersaDoc 4000 molecular imaging system (Bio-Rad Laboratories, Inc.) and NBD-triacylglycerol levels were quantified with Quantity One software (Bio-Rad Laboratories, Inc.). To distinguish between DGAT1 and DGAT2, cell extracts were incubated with a selective DGAT1 inhibitor (PF-04620110, Sigma) prior to DGAT assays being performed [16]. DGAT2 activity was the activity remaining in the presence of inhibitor. DGAT1 activity = Total DGAT activity − DGAT2 activity.

### Lipid analyses

Lipids from cell lysates were extracted from equal amounts of cellular protein (500–1000 μg) with chloroform/methanol (2:1, v/v). Neutral lipids were then separated by thin-layer chromatography with the solvent system hexane:ethyl ether:acetic acid (80:20:1, v/v/v). Lipids were visualized by charring with 10% cupric sulfate/8% phosphoric acid and heating to 180 °C. Lipid levels were quantified by densitometry.

### Silencing of calnexin in HEK-293T cells

HEK-293T packaging cells were transfected with psPAX2, pMD2.G and pLKO.1 plasmids containing two different calnexin shRNA sequences and a non-targeting control (NT). Media containing lentivirus was collected 24 and 48 h post-transfection and was pooled and filtered. Stable calnexin knockdown and non-targeted control cell lines were generated by transducing HEK-293T cells with media containing lentivirus and 8 μg/mL polybrene for 24 h. Transduced cells were selected with puromycin (2 μg/mL) and then used for experiments. The efficiency of calnexin knockdown was assessed by immunoblotting.

### Isolation of crude mitochondrial and microsomal fractions

Cells used for fractionation experiments were washed and resuspended in PBS (pH 7.4). Cells were disrupted by 20 passages through a 27-gauge needle followed by centrifugation for 2 min at 1,000 x *g* to pellet cellular debris and nuclei. The cell lysate was centrifuged for 10 min at 10,000 x *g* (4 °C) to pellet crude mitochondria (mitochondria and mitochondria-associated membranes (MAM)). Crude mitochondria were resuspended in a solution of 50 mM Tris-HCl (pH 7.4), 250 mM sucrose. The supernatant was centrifuged at 100,000 ×*g* for 60 min in a Beckman Ti-70.1 rotor at 4 °C to pellet microsomes, which were resuspended in 50 mM Tris-HCl (pH 7.4), 250 mM sucrose.

### Statistical analyses

Data are presented as mean ± S.D. unless otherwise indicated. Means were compared by analysis of variance (ANOVA) and Tukey test.

## Results

### Identification of DGAT2-interacting proteins using BioId

**Fig 1:**
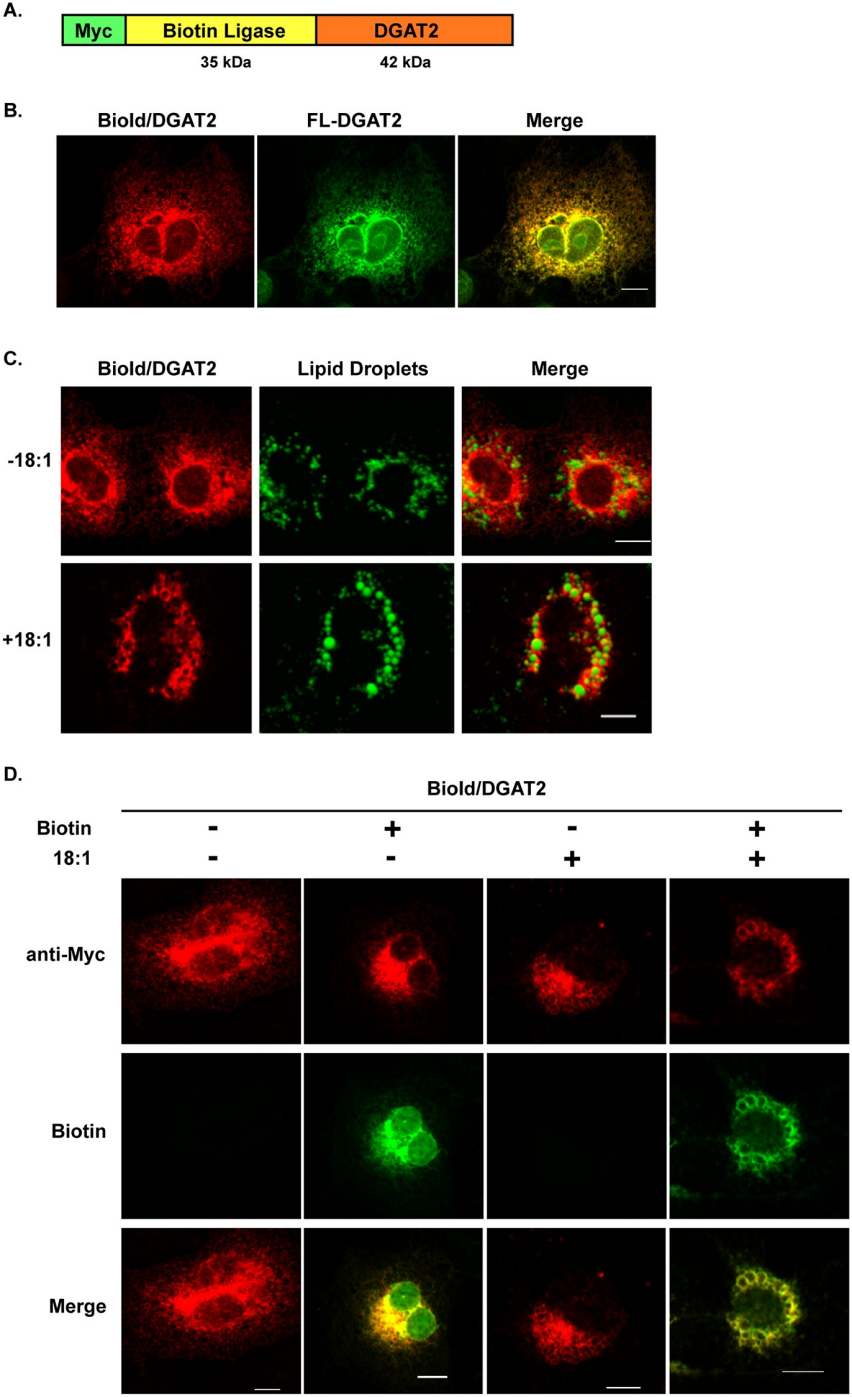
BioId/DGAT2 localizes to the ER and lipid droplets. (*A*) Murine DGAT2 was fused, in frame, to the C-terminus of Myc-tagged biotin ligase. The resulting fusion protein is ~ 77 kDa. Detection of BioId/DGAT2 by immunofluorescence microscopy. (*B*) COS-7 cells transfected with BioId/DGAT2 and was detected with anti-Myc (red). ER was visualized by co-transfection of cells with FL-DGAT2 (green). (*C*) Cells expressing BioId/DGAT2 (red) were treated with or without 0.5 mM oleate for 12 h. Lipid droplets were visualized with Bodipy 493/503 (green). (*D*) Detection of biotinylated proteins. COS-7 cells expressing BioId/DGAT2 were cultured as described in (*C*) with or without 50 μM biotin for 12 h. BioId/DGAT2 was detected with anti-Myc (red) and biotinylated proteins were detected with streptavidin-488 (green). Scale bar = 10 μm.

To identify proteins that interact with DGAT2, a BioId fusion protein was constructed where DGAT2 was fused in-frame to the C-terminus of a promiscuous biotin ligase, BirA* (Fig 1A) [12]. Expression of the BioId/DGAT2 fusion protein in COS-7 cells confirmed that DGAT2 was still targeted correctly. In the absence of oleate, BioId/DGAT2 was present in the ER where it co-localized with FL-DGAT2 (Fig 1B). When oleate was added to the culture media, BioId/DGAT2 was present around lipid droplets (Fig 1C). Furthermore, like DGAT2, BioId/DGAT2 stimulated the formation of large lipid droplets. Taken together, fusing DGAT2 to the C-terminus does not appear to change its localization or function in cells. This is consistent with experiments performed using an mCherry/DGAT2 fusion protein [4].

When biotin was added to the culture medium, with or without oleate, there was a strong biotinylation signal that co-localized with BioId/DGAT2 both in the ER and around lipid droplets (Fig 1D). No biotinylation signal was detected in cells not given exogenous biotin. These biotinylated proteins represent possible interacting proteins of DGAT2.

**Fig 2:**
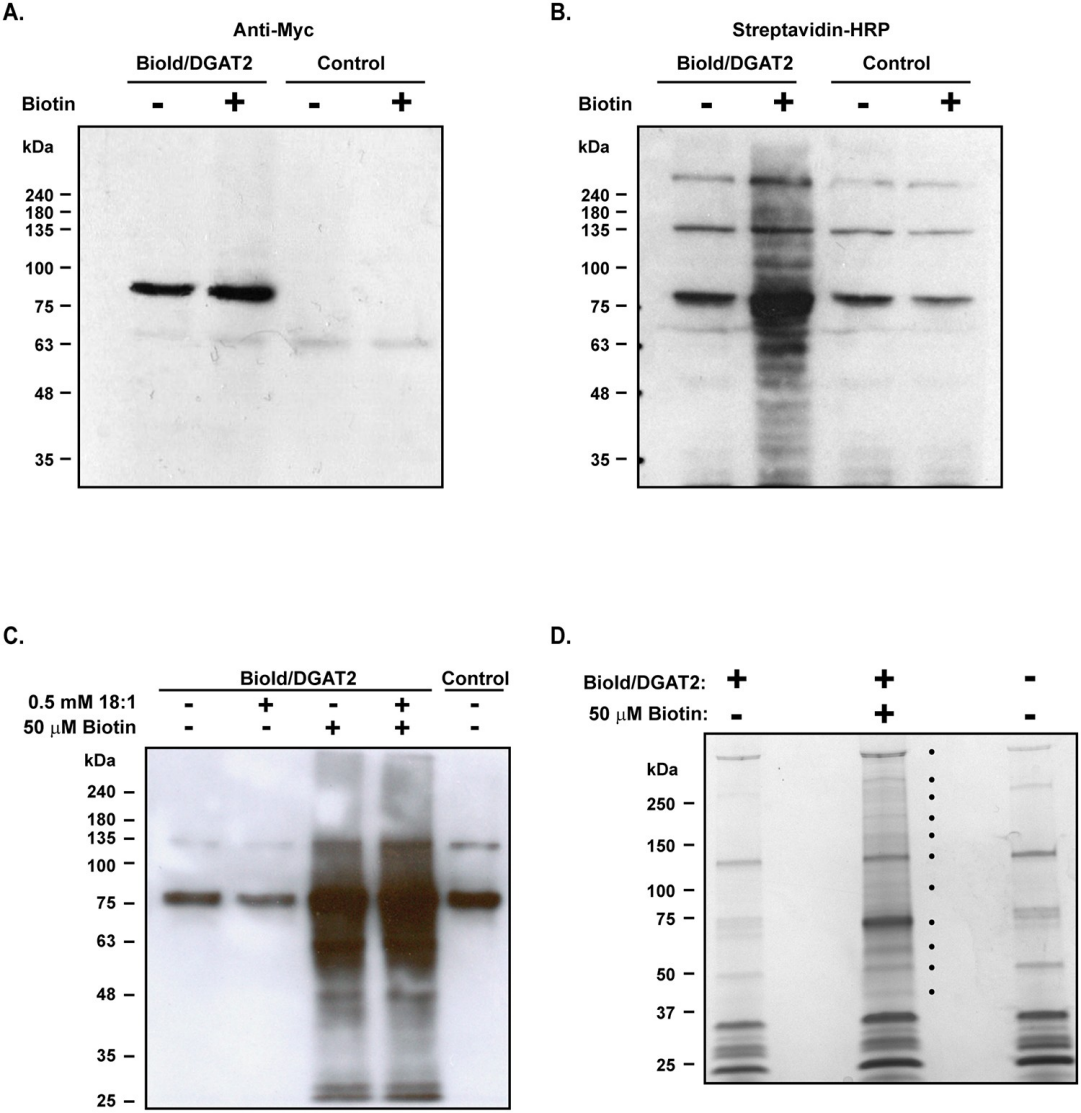
Affinity purification of proteins biotinylated by BioId/DGAT2. HEK-293T cells expressing BioId/DGAT2 were incubated with or without 50 μM biotin for 12 h. Cell lysates were immunoblotted with (*A*) anti-Myc and (*B*) streptavidin-HRP to detect BioId/DGAT2 and biotinylated proteins, respectively. Control: cells expressing LacZ. (*C*) HEK-293T cells expressing BioId/DGAT2 were incubated with or without 50 μM biotin and with or without 0.5 mM oleate for 12 h. Biotinylated proteins were captured with magnetic streptavidin beads, separated by SDS-PAGE and detected with streptavidin-HRP. (*D*) Separation of biotinylated proteins for in-gel digestion and mass spectrometry. Biotinylated proteins from (*C*) were stained with Bio-Safe Coomassie Brilliant Blue G-250 stain.

To identify possible DGAT2 interacting proteins, BioId/DGAT2 was expressed in HEK-293T cells. Expression was confirmed by immunoblotting with anti-Myc. The ~77 kDa fusion protein was detected in cells transfected with BioId/DGAT2, but not in untransfected cells (Fig 2A). When samples were immunoblotted with streptavidin-HRP, multiple biotinylated proteins were detected in BioId/DGAT2 expressing cell lysates that had been incubated with 50 μM biotin (Fig 2B). There was minimal biotinylation in untransfected cells or cells not exposed to exogenous biotin.

Biotinylated proteins were isolated from solubilized cell extracts using magnetic beads coupled to streptavidin. Several biotinylated proteins were detected in the streptavidin immunoprecipitates from cells expressing BioId/DGAT2 that had been incubated with biotin (Fig 2C). There was no obvious difference in the biotinylation pattern of cells treated with or without oleate. Biotinylated proteins from cells treated with oleate were separated by SDS-PAGE and stained with Coomassie blue (Fig 2D). Several unique proteins were observed in BioId/DGAT2 expressing cells treated with biotin, that were absent from the controls. These bands were excised, trypsinized and peptides were identified by mass spectrometry.

Mass spectrometry identified 512 unique BioId/DGAT2 proximal proteins that were not detected in the control samples (S1 Table). The candidate DGAT2 interactors were selected using the criteria of a false discovery rate of less than 1% and the detection of at least two unique peptides.

Several studies have established a comprehensive list of proteins present in the lipid droplet proteome. We compared these to our data set and identified 48 known lipid droplet proteins (~9%) (S2 Table). This observation provides additional evidence that DGAT2 interacts with lipid droplets and is proximal to proteins associated with this organelle. Interestingly, DGAT2 has not been identified in the numerous lipid droplet proteome studies from mammalian cells and tissues.

Analysis of Reactome pathways with WebGestalt (http://www.webgestalt.org/option.php) revealed that approximately 40 DGAT2-interacting proteins were overrepresented in both membrane trafficking (R-HSA-199991; P value = 3.37 x 10^−8^) and vesicle-mediated transport (R-HSA-5653656; P Value = 8.57 x 10^−9^) pathways (S3 and S4 Tables). Proteins involved in both COPI and COPII coatomer mediated protein trafficking were identified. Protein trafficking utilizing both pathways has been implicated in the movement of proteins to lipid droplets [17–19].

We also identified 21 DGAT2 interactors that were overrepresented in the KEGG protein processing pathways in the ER (HSA-04141; P value = 6.75e-09) (S5 Table). A cluster of proteins in this network, that includes HSPA1A, DNAJB1, HSPH1, NGLY1, NPLOC4 and VCP, are involved in ER-associated degradation. This is consistent with the findings that DGAT2 is degraded via the proteasome in a ubiquitin-dependent manner [20, 21].

### Calnexin interacts with DGAT2

Our BioId screen identified calnexin as a DGAT2 interacting protein (Table 1). Calnexin, in addition to calreticulin, are ER chaperones that were also overrepresented in the KEGG pathway described above. Calnexin assists in the proper folding of glycoproteins in the ER and modulates calcium homeostasis [22]. Calnexin was of interest as it is an integral membrane protein that, like DGAT2, has been found to be associated with lipid droplets and is enriched in mitochondrial associated membranes [2, 23, 24].

**Table 1:** Calnexin (Q16094) peptides identified by BioId/DGAT2 biotinylation.

Sequence	Observed Mass	Actual MH^+^	Charge	Peptide Score
(K)AEEDEILNR(S)	544.766	1088.522	2	16.3
(K)TGIYEEK(H)	420.211	839.414	2	13.7
(R)EIEDPEDR(K)	501.723	1002.437	2	15.7
(R)KPEDWDERPK(I)	433.884	1299.633	3	15.4
(R)PVIDNPNYK(G)	530.278	1059.547	2	13.5
(R)CESAPGCGVWQR(P)	703.802	1406.594	2	9.3
(K)AADGAAEPGVVGQ(M)	571.278	1141.548	2	8.5
(K)RPDADLK(T)	407.726	814.442	2	8.4
(K)TYFTDK(K)	387.6887	774.367	2	12.1
(K)TDAPQPDVK(E)	485.7463	970.484	3	11.9
(K)AKKDDTDDEIAK(Y)	450.2275	1348.659	3	9.2
(K)GLVLMSR(A)	388.2291	775.449	2	11.4
(K)TPELNLDQFHDK(T)	486.2428	1456.707	3	10.7
(K)HHAISAK(L)	382.2144	763.421	2	10.1
(K)AEEDEILNRSPR(N)	503.5648	1428.708	3	13.7

**Fig 3:**
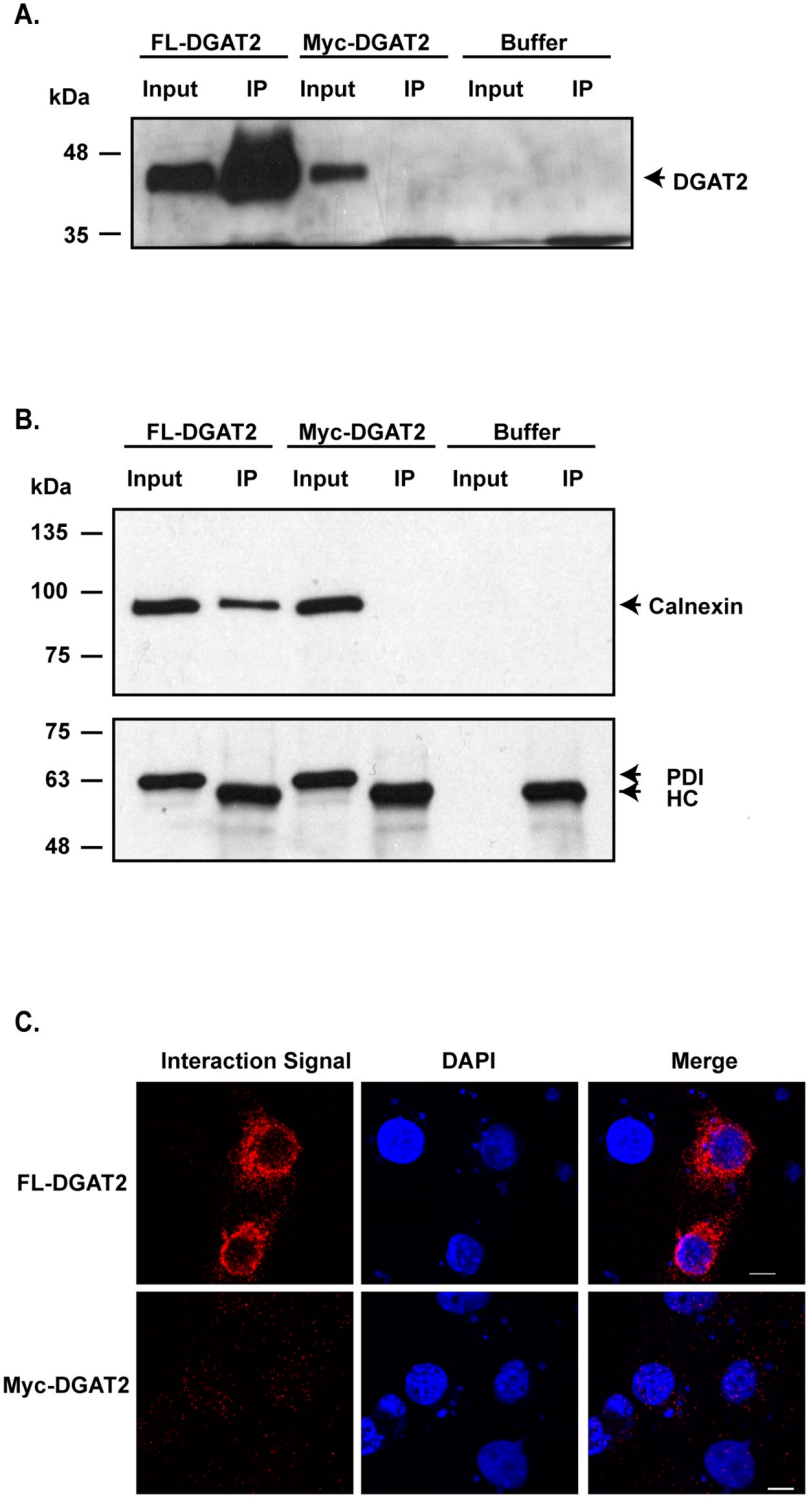
Identification of calnexin as a DGAT2 interacting protein by co-immunoprecipitation and mass spectrometry. (*A*) HEK-293T cells were transfected with either FL-DGAT2 or myc-DGAT2. FL-DGAT2 was immunoprecipitated with anti-FLAG agarose from detergent solubilized material. Immunoprecipitates (IP) were separated by SDS-PAGE and were then probed with anti-DGAT2. (*B*) Calnexin, but not PDI, was detected in anti-FLAG immunoprecipitates by immunoblotting. HC; heavy chain. (*C*) Interaction of DGAT2 and calnexin was detected *in situ* using a proximity ligation assay. COS-7 cells expressing either FL-DGAT2 or Myc-DGAT2 were stained with mouse anti-FLAG and rabbit anti-calnexin antibodies. Interaction signals (red) were detected using a Duolink detection kit. Nuclei were stained with DAPI (blue). Scale bar = 10 μm.

The interaction of DGAT2 with calnexin was confirmed by two additional independent methods. Using co-immunoprecipitation and mass spectrometry, six unique calnexin peptides were identified in FL-DGAT2 immunoprecipitates from HEK-293T cells expressing FL-DGAT2 (Fig 3A and Table 2). Calnexin was not identified in the control sample, which was an anti-FLAG immunoprecipitate of Myc-DGAT2 expressed in HEK-293T cells. Calnexin was also detected with an anti-calnexin antibody in the anti-FLAG immunoprecipitates from cells expressing FL-DGAT2, but not Myc-DGAT2 (Fig 3B). Unlike calnexin, another resident ER protein, protein disulfide isomerase, was not detected in the anti-FLAG immunoprecipitates.

**Table 2:** Calnexin (Q16094) peptides identified by co-immunoprecipitation of DGAT2.

Sequence	Observed Mass	Actual MH^+^	Charge	Peptide Score
(K)AEEDEILNR(S)	544.7694	1088.522	2	15.3
(R)KIPNPDFFEDLEPFR(M)	621.9829	1863.928	3	13.7
(R)IVDDWANDGWGLK(K)	744.8631	1488.712	2	18.2
(K)IPDPEAVKPDDWDEDAPAK(I)	703.3335	2107.982	3	18.3
(R)GTLSGWILSK(A)	531.3033	1061.599	2	18.2
(K)APVPTGEVYFADSFDR(G)	590.9529	1770.833	3	16.7

The DGAT2/calnexin interaction was also confirmed *in situ* using a proximity ligation assay [25]. COS-7 cells expressing either FL-DGAT2 or Myc-DGAT2 were fixed, permeabilized and then incubated with mouse anti-FLAG and rabbit anti-calnexin antibodies. Modified secondary antibody probes were then added that will interact if they are in close proximity, producing a red fluorescent signal. A fluorescent interaction signal was only detected when FL-DGAT2, and not Myc-DGAT2, was expressed, indicating that DGAT2 and calnexin were in close proximity to each other (< 40 nm) (Fig 3C). These data confirm our BioId and co-immunoprecipitation experiments and provide additional evidence that DGAT2 and calnexin interact in intact cells.

### Calnexin levels increase during adipocyte differentiation

**Fig 4:**
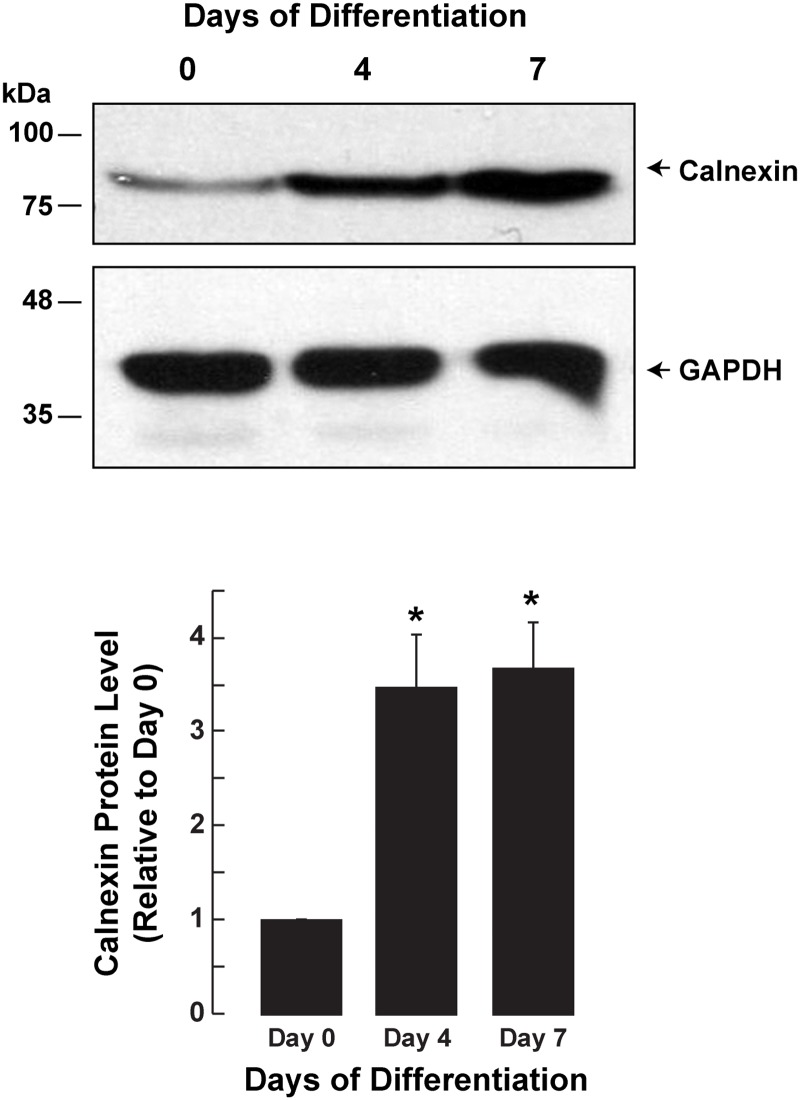
Calnexin protein abundance during mouse 3T3-L1 adipocyte differentiation. Cell lysates were prepared from 3T3-L1 cells at different days of adipocyte differentiation. Protein samples were separated by SDS-PAGE and immunoblotted for calnexin and GAPDH. Data are the mean of three experiments. *, *p*<0.01, Day 0 *versus* Days 4 and 7.

The interaction between calnexin and DGAT2 suggested that calnexin may have an under-appreciated role in lipid metabolism, including adipocyte differentiation. The differentiation of 3T3-L1 cells into adipocytes involves significant changes in gene expression, lipid and membrane synthesis and the cellular proteome [26–29]. We examined calnexin abundance during adipocyte differentiation. After 7 days of differentiation calnexin protein levels increased ~4-fold (Fig 4).

### Cells lacking calnexin have altered lipid droplet morphology

**Fig 5:**
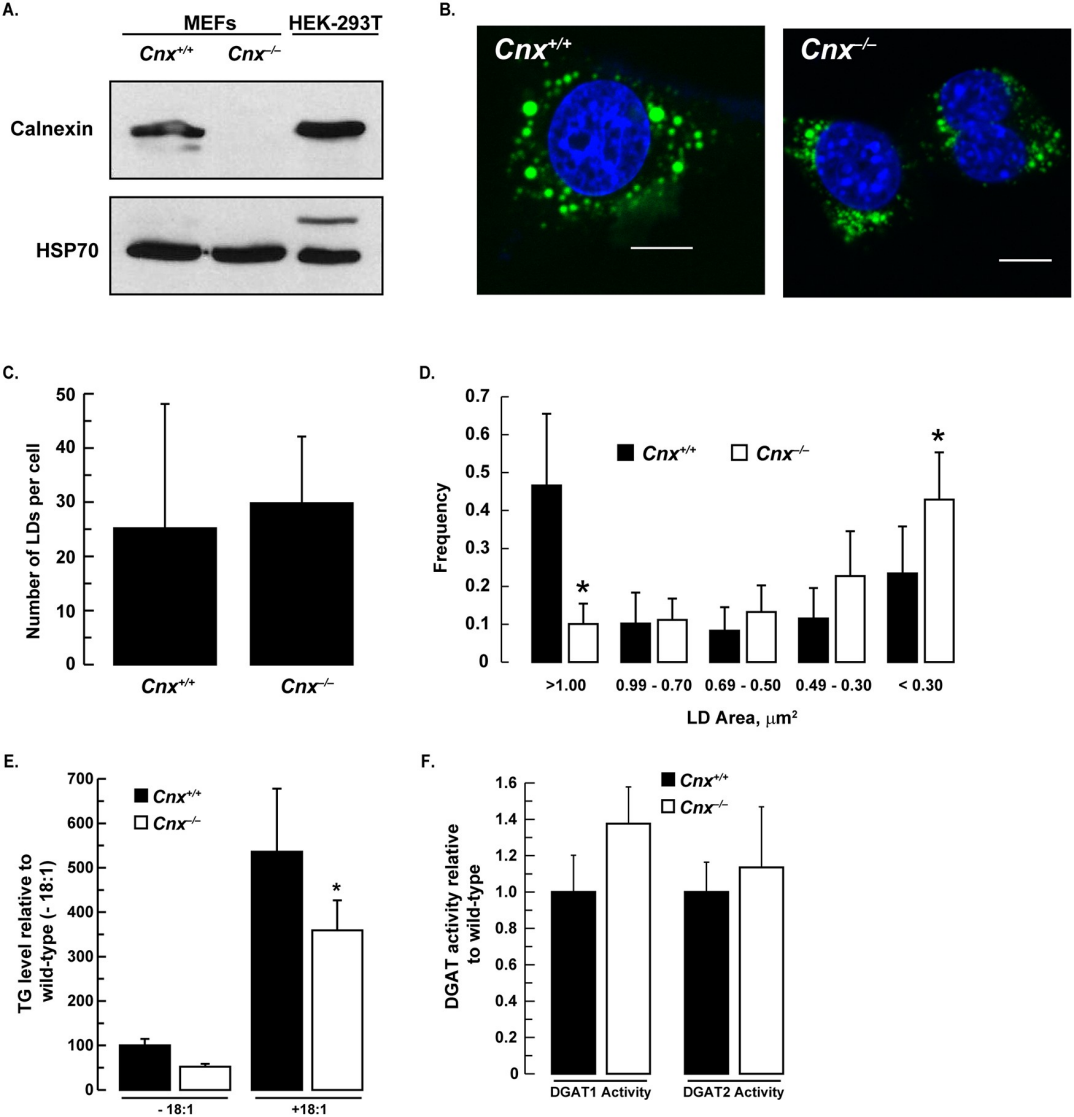
Reduced lipid droplet size in calnexin-deficient MEFs. (*A*) Immunoblot showing that calnexin was not detectible in *Cnx2*^*–/–*^ MEFs. (*B*) *Cnx2*^*+/+*^ and *Cnx2*^*–/–*^ MEFs were incubated with 0.5 mM oleate for 12 h and then stained with Bodipy 493/503 and DAPI. Scale bars, 10 μm. Lipid droplet number (*C*) and area (*D*) were quantified using ImageJ (National Institutes of Health, rsb.info.nih.gov/ij). *, *p*<0.001, *Cnx2*^*+/+*^ *versus Cnx2*^*–/–*^ MEFs. Mean lipid droplet number per cell and lipid droplet area were calculated from 17 to 25 cells. (*E*) Lipids were extracted from *Cnx2*^*+/+*^ and *Cnx2*^*–/–*^ MEFs treated with or without 0.5 mM oleate for 12 h. Data are the mean of three experiments performed in duplicate. *, *p*<0.001, *Cnx2*^*+/+*^ *versus Cnx2*^*–/–*^ oleate-loaded MEFs. (*F*) *In vitro* DGAT1 and DGAT2 activities from *Cnx2*^*+/+*^ and *Cnx2*^*–/–*^ cell extracts. Data are the mean of two experiments performed in triplicate.

To determine if the ER chaperone has a role in lipid droplet formation, wild-type (*Cnx*^*+/+*^) mouse embryonic fibroblasts (MEFs) and calnexin-deficient MEFs (*Cnx*^*–/–*^) (Fig 5A) [11] were incubated with 0.5 mM oleate to stimulate lipid droplet formation. While there was no difference in total lipid droplet number, there was a noticeable absence of large lipid droplets (>3 μm^2^ area) in *Cnx*^*–/–*^ cells (Fig 5B–5D). Instead, these cells had an increased number of smaller lipid droplets (<0.3 μm^2^ area).

The decrease in lipid droplet size suggested that the TG content of cells lacking calnexin would be reduced as well. Indeed, calnexin-deficient cells had a ~ 33% reduction in TG levels compared to wild-type cells cultured with 0.5 mM oleate (Fig 5E). The decrease in intracellular levels of TG could not be accounted for by altered DGAT activity. *In vitro* DGAT1 and DGAT2 activities were not affected by the absence of calnexin (Fig 5F).

### The subcellular localization and stability of DGAT2 are not altered by the absence of calnexin

**Fig 6:**
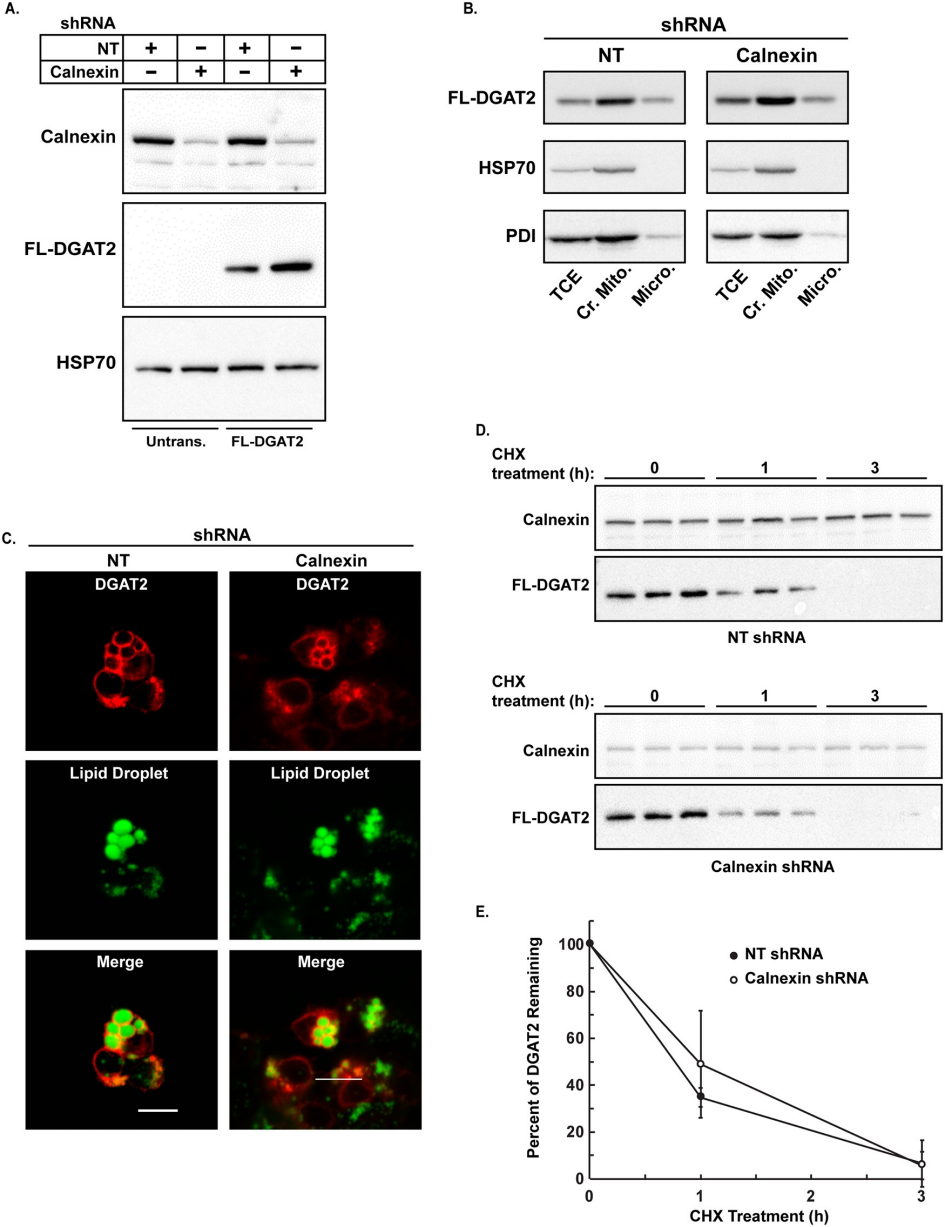
The subcellular localization and stability of DGAT2 is not altered in the absence of calnexin. (*A*) Immunoblot with anti-calnexin showing the efficient silencing of calnexin in HEK-293T cells with shRNAs (top panel). The control (NT) refers to HEK-293T cells transduced with a non-targeting shRNA. The bottom panel shows non-targeted and calnexin knockdown cells transiently transfected with FL-DGAT2 (2 right lanes). Untransfected cells (Untrans.) are the 2 left lanes. (*B*) Total cell extracts (TCE), crude mitochondria (Cr. Mito.) and microsomes (Micro.) were separated by SDS-PAGE and immunoblotted with anti-FLAG, anti-PDI and HSP70 antibodies. (*C*) Non-targeted and calnexin knockdown cells were transfected with FL-DGAT2 and treated with 0.5 mM oleate for 12 h. After fixation and permeabilization, cells were stained with anti-FLAG and BODIPY 493/503 to visualize lipid droplets. Scale bars: 10 μm. (*D*) 100 μg/mL CHX was added to the culture medium of HEK-293T (non-targeted and calnexin knockdown) cells expressing FL-DGAT2. Cells were harvested 0, 1 and 3 h after the addition of CHX. The amount of FL-DGAT2 and calnexin present after CHX treatment was determined by immunoblotting. (*E*) Quantification of the data in Fig 6E. Data are the mean of three independent experiments, performed in triplicate.

Calnexin may function as a chaperone for DGAT2 facilitating its localization to MAM and/or lipid droplets. To test this, calnexin was silenced in HEK-293T cells using RNA interference. HEK-293T cells transduced with shRNAs for calnexin led to an ~80% decrease in calnexin abundance, compared to the non-targeted control (Fig 6A). Subcellular fraction experiments showed that DGAT2 was enriched in the crude mitochondrial fraction relative to the microsomal fraction, indicating that its localization to MAM is not calnexin-dependent (Fig 6B). Similarly, DGAT2 was localized to lipid droplets in calnexin knockdown cells (Fig 6C).

DGAT2 is rapidly degraded in cells by the proteasome in a ubiquitin-dependent manner [21]. We observed that FL-DGAT2 was present at higher levels in calnexin knockdown cells suggesting that DGAT2 degradation may be impaired in the absence of calnexin (Fig 6A and 6B). To determine if calnexin has a role in DGAT2 degradation, we examined DGAT2 stability in calnexin knockdown cells. After blocking new protein synthesis with cycloheximide, we found no difference in the rate of DGAT2 degradation in the presence or absence of calnexin (Fig 6D and 6E).

## Discussion

In this study, proximity-dependent biotin labeling (BioId) was used to identify proteins that interact with DGAT2. Of the candidate DGAT2-interacting proteins identified, calnexin was of interest as it is an integral membrane protein that, like DGAT2, has been found to be associated with lipid droplets and is enriched in MAM [2, 23, 24, 30–33]. MAM is a specialized ER subdomain in physical contact with the outer mitochondrial membrane. This close apposition of membranes is believed to facilitate the intracellular transport of lipids between the ER and mitochondria [34–36]. The calnexin-DGAT2 interaction was confirmed by co-immunoprecipitation and proximity ligation assay.

Calnexin is most well-known for assisting in the proper folding of glycoproteins in the ER [22]. It also monitors the assembly of transmembrane domains in the ER membrane and binds to misfolded regions in a glycan-independent manner [37]. Calnexin has more recently been proposed to have functions unrelated to protein folding as it interacts with several cytoplasmic proteins involved in signaling and lipid metabolism [38–40]. For example, PTP-1B is a tyrosine phosphatase that is tightly bound to the ER membrane via a hydrophobic domain reminiscent of DGAT2 [3, 41]. The interaction of PTP-1B with the ER is dependent on calnexin [40]. Interestingly, PTP-1B was present in our BioId/DGAT2 data set providing additional evidence that DGAT2 is found in close proximity to calnexin.

Calnexin also interacts with a cytosolic *N*-myristoyltransferase retaining it at the ER membrane [39]. The co-translational attachment of myristate to the N-terminal glycine of a protein has been implicated in a diverse array of processes including cell signaling, protein-membrane and protein-protein interactions [42, 43].

The interaction of calnexin with DGAT2 suggested that this ER chaperone has a role in modulating DGAT2 function, especially since they are both localized to MAM and lipid droplets. Our experiments showed that in the absence of calnexin, the number of large lipid droplets in mouse embryonic fibroblasts was decreased, with a corresponding decrease in intracellular TG. However, DGAT2 activity was not altered, suggesting that calnexin does not directly modulate DGAT2 activity. Our experiments to show that calnexin had no role in localizing DGAT2 to lipid droplets and MAM, or in DGAT2 degradation, were also negative. However, it is possible that other ER chaperones partially compensate for the absence of calnexin. Human T lymphoblastoid cell lines lacking calnexin have modest increases in BiP and calreticulin protein levels [44]. Both of these proteins were also identified as possible DGAT2 interactors (Table 1).

The changes in TG metabolism and lipid droplet morphology that we observed in *Cnx2*^*–/–*^ MEFs may have been caused by the accumulation of misfolded proteins [11]. *Cnx2*^*–/–*^ MEFs have increased constitutively active ER stress and proteasomal activity which most likely results in the chronic alteration of metabolic pathways resulting in decreased TG levels [11]. In adipocytes, pharmacological induction of ER stress activated lipolysis with a corresponding reduction in lipid droplet size [45, 46]. Alternatively, the absence of calnexin may have affected the biochemical reactions of the TG biosynthetic pathway upstream of DGAT2 leading to reduced TG synthesis.

Unfortunately, we were not able to determine the significance of the interaction of calnexin with DGAT2 in this study. Going forward, it will be important to map the regions of DGAT2 and calnexin that interact, since most of DGAT2 is present in the cytosol, while most of calnexin is in the ER lumen [3, 4, 47]. It will also be important to determine if the dynamics of this interaction is altered during oleate-stimulated lipid synthesis. This is something that our experiments did not address. In conclusion, calnexin is dispensable for lipid droplet formation, although in its absence lipid droplet morphology is altered.

## Supporting information

S1 Table DGAT2/BioId Interactors. (XLSX)

S2 Table Lipid droplet proteins identified by BioId/DGAT2 biotinylation. (DOCX)

S3 Table Overrepresentation of DGAT2-interacting proteins in Reactome pathway HSA-199991 (Membrane trafficking). (DOCX)

S4 Table Overrepresentation of DGAT2-interacting proteins in Reactome pathway HSA-5653656 (Vesicle-mediated transport). (DOCX)

S5 Table Overrepresentation of DGAT2-interacting proteins in KEGG pathway HAS-04141 (protein processing in endoplasmic reticulum). (DOCX)
