# Supplementary material for: Identification of calnexin as a diacylglycerol acyltransferase-2 interacting protein
Source: PLoS One. 2019 Jan 7;14(1):e0210396. doi: 10.1371/journal.pone.0210396 (PMC6322727; doi:10.1371/journal.pone.0210396)
Supplement: S2 Table — (DOCX) [file pone.0210396.s002.docx]

**Table S2. Lipid droplet proteins identified by BioID/DGAT2 biotinylation**

| **Gene** | **Protein Name** | **Reference** |
| --- | --- | --- |
| ACBD3 | Golgi resident protein GCP60 (ACBD3) | 1 |
| ACSL3 | Long-chain acyl-CoA synthetase 3 | 2-6 |
| AKAP1 | A-kinase anchor protein | 1 |
| ALDH3 | Fatty aldehyde dehydrogenase | 7,8 |
| ARFGAP1 | ADP-ribosylation factor GTPase-activating protein 1 | 9,10 |
| ATG2B | Autophagy-related protein 2 homolog B | 1 |
| AUP1 | Ancient ubiquitous protein 1 | 4,5,11-14 |
| CALR | Calreticulin | 14,15 |
| CANX | Calnexin | 4-6,12,14-16 |
| CCT2 | T-complex protein 1 subunit beta (TCP-1-beta) | 1 |
| CCT4 | T-complex protein 1 subunit delta (TCP-1-delta) | 1 |
| CCT7 | T-complex protein 1 subunit eta (TCP-1-eta) | 1 |
| CCT8 | T-complex protein 1 subunit theta (TCP-1-theta) | 1 |
| COPB' | Coatomer subunit beta' | 1 |
| COPB1 | Coatomer subunit beta | 4,17,18 |
| CSE1L | Exportin-2 | 19 |
| CYP51A1 | Lanosterol 14-alpha demethylase | 1 |
| DNM1L | Dynamin-1-like protein | 6 |
| EEF2 | Elongation factor 2 | 1 |
| ESYT1 | Extended synaptotagmin-1 | 20 |
| FAF2 | FAS-associated factor 2 (UBXD8) | 4-6,14,21-23 |
| GANAB | Neutral alpha-glucosidase AB | 12,14 |
| GCN1 | eIF-2-alpha kinase activator GCN1 | 1 |
| HSPA1A | Heat shock 70 kDa protein 1A | 14 |
| HSPA5 | Endoplasmic reticulum chaperone BiP (GRP78) | 4-6,12-14,16 |
| KPNB1 | Importin subunit beta-1 | 14 |
| MAN2A1 | Alpha-mannosidase 2 | 1 |
| NSDHL | Sterol-4-alpha-carboxylate 3-dehydrogenase | 1 |
| NUP93 | Nuclear pore complex protein Nup93 | 1 |
| NUP98 | Nuclear pore complex protein Nup98-Nup96 | 1 |
| PLIN2 | Perilipin-2 | 4-6,12-16 |
| POR | NADPH cytochrome P450 reductase | 14 |
| PRKCSH | Glucosidase 2 beta | 20 |
| PSMD2 | 26S proteasome non-ATPase regulatory subunit 2 | 4,5,16 |
| RAB15 | Ras-related protein Rab-15 | 14 |
| RAB3GAP2 | Rab3 GTPase-activating protein non-catalytic subunit | 10 |
| RINT1 | RAD50-interacting protein 1 | 1 |
| SLC3A2 | 4F2 cell-surface antigen heavy chain | 1 |
| SPART | Spartin | 1 |
| SRP68 | Signal recognition particle subunit SRP68 | 1 |
| STIM1 | Stromal interaction molecule 1 | 1 |
| TRIM27 | Zinc finger protein RFP | 1 |
| UBXN4 | UBX domain-containing protein 4 | 4,14,23 |
| UFL1 | E3 UFM1-protein ligase 1 | 1 |
| VCP | Transitional endoplasmic reticulum ATPase (VCP) | 6,22,23 |
| VIM | Vimentin | 4,5,12,13,16,24 |
| VPS13C | Vacuolar protein sorting-associated protein 13C | 6 |

**References for Table 1**

1. Wang, W., Wei, S., Li, L., Su, X., Du, C., Li, F., Geng, B., Liu, P., and Xu, G. (2015) Proteomic analysis of murine testes lipid droplets. *Scientific reports* **5**, 12070

2. Wilfling, F., Wang, H., Haas, J. T., Krahmer, N., Gould, T. J., Uchida, A., Cheng, J., Graham, M., Christiano, R., Fröhlich, F., Liu, X., Buhman, K. K., Coleman, R. A., Bewersdorf, J., Farese Jr, R. V., and Walther, T. C. (2013) Triacylglycerol synthesis enzymes mediate lipid droplet growth by relocalizing from the ER to lipid droplets. *Developmental Cell* **24**, 384-399

3. Poppelreuther, M., Rudolph, B., Du, C., Großmann, R., Becker, M., Thiele, C., Ehehalt, R., and Füllekrug, J. (2012) The N-terminal region of acyl-CoA synthetase 3 is essential for both the localization on lipid droplets and the function in fatty acid uptake. *J. Lipid Res.* **53**, 888-900

4. Bartz, R., Zehmer, J. K., Zhu, M., Chen, Y., Serrero, G., Zhao, Y., and Liu, P. (2007) Dynamic Activity of Lipid Droplets: Protein Phosphorylation and GTP-Mediated Protein Translocation. *J. Proteome Res.* **6**, 3256-3265

5. Brasaemle, D. L., Dolios, G., Shapiro, L., and Wang, R. (2004) Proteomic analysis of proteins associated with lipid droplets of basal and lipolytically stimulated 3T3-L1 adipocytes. *J. Biol. Chem.* **279**, 46835–46842

6. Zhang, H., Wang, Y., Li, J., Yu, J., Pu, J., Li, L., Zhang, H., Zhang, S., Peng, G., Yang, F., and Liu, P. (2011) Proteome of Skeletal Muscle Lipid Droplet Reveals Association with Mitochondria and Apolipoprotein A-I. *Journal of Proteome Research* **10**, 4757-4768

7. Natter, K., Leitner, P., Faschinger, A., Wolinski, H., McCraith, S., Fields, S., and Kohlwein, S. D. (2005) The Spatial Organization of Lipid Synthesis in the Yeast <em>Saccharomyces cerevisiae</em> Derived from Large Scale Green Fluorescent Protein Tagging and High Resolution Microscopy. *Molecular &amp; Cellular Proteomics* **4**, 662-672

8. Currie, E., Guo, X., Christiano, R., Chitraju, C., Kory, N., Harrison, K., Haas, J., Walther, T. C., and Farese, R. V. (2014) High confidence proteomic analysis of yeast LDs identifies additional droplet proteins and reveals connections to dolichol synthesis and sterol acetylation. *J. Lipid Res.* **55**, 1465-1477

9. Gannon, J., Fernandez-Rodriguez, J., Alamri, H., Feng, S. B., Kalantari, F., Negi, S., Wong, A. H. Y., Mazur, A., Asp, L., Fazel, A., Salman, A., Lazaris, A., Metrakos, P., Bergeron, J. J. M., and Nilsson, T. (2014) ARFGAP1 Is Dynamically Associated with Lipid Droplets in Hepatocytes. *PLOS ONE* **9**, e111309

10. Bersuker, K., Peterson, C. W. H., To, M., Sahl, S. J., Savikhin, V., Grossman, E. A., Nomura, D. K., and Olzmann, J. A. (2018) A Proximity Labeling Strategy Provides Insights into the Composition and Dynamics of Lipid Droplet Proteomes. *Developmental Cell* **44**, 97-112.e117

11. Jo, Y., Hartman, I. Z., and DeBose-Boyd, R. A. (2013) Ancient ubiquitous protein-1 mediates sterol-induced ubiquitination of 3-hydroxy-3-methylglutaryl CoA reductase in lipid droplet–associated endoplasmic reticulum membranes. *Mol. Biol. Cell* **24**, 169-183

12. Khor, V. K., Ahrends, R., Lin, Y., Shen, W.-J., Adams, C. M., Roseman, A. N., Cortez, Y., Teruel, M. N., Azhar, S., and Kraemer, F. B. (2014) The Proteome of Cholesteryl-Ester-Enriched Versus Triacylglycerol-Enriched Lipid Droplets. *PLOS ONE* **9**, e105047

13. Liu, P., Ying, Y., Zhao, Y., Mundy, D. I., Zhu, M., and Anderson, R. G. (2004) Chinese hamster ovary K2 cell lipid droplets appear to be metabolic organelles involved in membrane traffic. *J. Biol. Chem.* **279**, 3787–3792

14. Bouchoux, J., Beilstein, F., Pauquai, T., Guerrera, I. C., Chateau, D., Ly, N., Alqub, M., Klein, C., Chambaz, J., Rousset, M., Lacorte, J.-M., Morel, E., and Demignot, S. (2011) The proteome of cytosolic lipid droplets isolated from differentiated Caco-2/TC7 enterocytes reveals cell-specific characteristics. *Biol. Cell* **103**, 499-517

15. Turro, S., Ingelmo-Torres, M., Estanyol, J. M., Tebar, F., Fernandez, M. A., Albor, C. V., Gaus, K., Grewal, T., Enrich, C., and Pol, A. (2006) Identification and Characterization of Associated with Lipid Droplet Protein 1: A Novel Membrane-Associated Protein That Resides on Hepatic Lipid Droplets. *Traffic* **7**, 1254-1269

16. Cho, S. Y., Shin, E. S., Park, P. J., Shin, D. W., Chang, H. K., Kim, D., Lee, H. H., Lee, J. H., Kim, S. H., Song, M. J., Chang, I.-S., Lee, O. S., and Lee, T. R. (2007) Identification of Mouse Prp19p as a Lipid Droplet-associated Protein and Its Possible Involvement in the Biogenesis of Lipid Droplets. *J. Biol. Chem.* **282**, 2456-2465

17. Wilfling, F., Thiam, A. R., Olarte, M.-J., Wang, J., Beck, R., Gould, T. J., Allgeyer, E. S., Pincet, F., Bewersdorf, J., Farese, R. V., Jr., and Walther, T. C. (2014) Arf1/COPI machinery acts directly on lipid droplets and enables their connection to the ER for protein targeting. *eLife* **3**, e01607

18. Soni, K. G., Mardones, G. A., Sougrat, R., Smirnova, E., Jackson, C. L., and Bonifacino, J. S. (2009) Coatomer-dependent protein delivery to lipid droplets. *J. Cell Sci.* **122**, 1834-1841

19. Dahlhoff, M., Fröhlich, T., Arnold, G. J., Müller, U., Leonhardt, H., Zouboulis, C. C., and Schneider, M. R. (2015) Characterization of the sebocyte lipid droplet proteome reveals novel potential regulators of sebaceous lipogenesis. *Exp. Cell Res.* **332**, 146-155

20. Ding, Y., Wu, Y., Zeng, R., and Liao, K. (2012) Proteomic profiling of lipid droplet-associated proteins in primary adipocytes of normal and obese mouse. *Acta Biochimica et Biophysica Sinica* **44**, 394-406

21. Zehmer, J. K., Bartz, R., Bisel, B., Liu, P., Seemann, J., and Anderson, R. G. W. (2009) Targeting sequences of UBXD8 and AAM-B reveal that the ER has a direct role in the emergence and regression of lipid droplets. *J. Cell Sci.* **122**, 3694-3702

22. Olzmann, J. A., Richter, C. M., and Kopito, R. R. (2013) Spatial regulation of UBXD8 and p97/VCP controls ATGL-mediated lipid droplet turnover. *Proceedings of the National Academy of Sciences* **110**, 1345-1350

23. Suzuki, M., Otsuka, T., Ohsaki, Y., Cheng, J., Taniguchi, T., Hashimoto, H., Taniguchi, H., and Fujimoto, T. (2012) Derlin-1 and UBXD8 are engaged in dislocation and degradation of lipidated ApoB-100 at lipid droplets. *Mol. Biol. Cell* **23**, 800-810

24. Heid, H., Rickelt, S., Zimbelmann, R., Winter, S., Schumacher, H., Dörflinger, Y., Kuhn, C., and Franke, W. W. (2014) On the Formation of Lipid Droplets in Human Adipocytes: The Organization of the Perilipin–Vimentin Cortex. *PLOS ONE* **9**, e90386
