# Supplementary material for: Identification of calnexin as a diacylglycerol acyltransferase-2 interacting protein
Source: PLoS One. 2019 Jan 7;14(1):e0210396. doi: 10.1371/journal.pone.0210396 (PMC6322727; doi:10.1371/journal.pone.0210396)
Supplement: S4 Table — (DOCX) [file pone.0210396.s004.docx]

**Table S4. Overrepresentation of DGAT2-interacting proteins in Reactome pathway** **HSA-5653656 (Vesicle-mediated transport)**

| **C=662; O=47; E=19.27; R=2.44; PValue=8.57e-09; FDR=1.37e-05** |  |  |  |
| --- | --- | --- | --- |
|  |  |  |  |
| **UserID** | **Gene Symbol** | **Gene Name** | **Entrez Gene** |
| O95235 | KIF20A | kinesin family member 20A | 10112 |
| Q9HCU5 | PREB | prolactin regulatory element binding | 10113 |
| O95487 | SEC24B | SEC24 homolog B, COPII coat complex component | 10427 |
| Q92598 | HSPH1 | heat shock protein family H (Hsp110) member 1 | 10808 |
| P26374 | CHML | CHM like, Rab escort protein 2 | 1122 |
| P53618 | COPB1 | coatomer protein complex subunit beta 1 | 1315 |
| Q14204 | DYNC1H1 | dynein cytoplasmic 1 heavy chain 1 | 1778 |
| O14641 | DVL2 | dishevelled segment polarity protein 2 | 1856 |
| Q9UPN7 | PPP6R1 | protein phosphatase 6 regulatory subunit 1 | 22870 |
| Q9Y2K3 | MYH15 | myosin heavy chain 15 | 22989 |
| O60333 | KIF1B | kinesin family member 1B | 23095 |
| Q9NZ52 | GGA3 | golgi associated, gamma adaptin ear containing, ARF binding protein 3 | 23163 |
| P02794 | FTH1 | ferritin heavy chain 1 | 2495 |
| P02792 | FTL | ferritin light chain | 2512 |
| Q9H2M9 | RAB3GAP2 | RAB3 GTPase activating non-catalytic protein subunit 2 | 25782 |
| Q14C86 | GAPVD1 | GTPase activating protein and VPS9 domains 1 | 26130 |
| Q9Y5X3 | SNX5 | sorting nexin 5 | 27131 |
| Q08379 | GOLGA2 | golgin A2 | 2801 |
| Q14789 | GOLGB1 | golgin B1 | 2804 |
| Q12955 | ANK3 | ankyrin 3 | 288 |
| P04114 | APOB | apolipoprotein B | 338 |
| P11717 | IGF2R | insulin like growth factor 2 receptor | 3482 |
| Q5JRA6 | MIA3 | MIA family member 3, ER export factor | 375056 |
| P52732 | KIF11 | kinesin family member 11 | 3832 |
| P49257 | LMAN1 | lectin, mannose binding 1 | 3998 |
| Q16706 | MAN2A1 | mannosidase alpha class 2A member 1 | 4124 |
| Q15843 | NEDD8 | neural precursor cell expressed, developmentally down-regulated 8 | 4738 |
| Q9NRC6 | SPTBN5 | spectrin beta, non-erythrocytic 5 | 51332 |
| Q96L93 | KIF16B | kinesin family member 16B | 55614 |
| Q8N6T3 | ARFGAP1 | ADP ribosylation factor GTPase activating protein 1 | 55738 |
| Q6NUQ1 | RINT1 | RAD50 interactor 1 | 60561 |
| Q9H3P7 | ACBD3 | acyl-CoA binding domain containing 3 | 64746 |
| Q13190 | STX5 | syntaxin 5 | 6811 |
| Q92574 | TSC1 | tuberous sclerosis 1 | 7248 |
| P0CG48 | UBC | ubiquitin C | 7316 |
| P62258 | YWHAE | tyrosine 3-monooxygenase | 7531 |
| Q5KU26 | COLEC12 | collectin subfamily member 12 | 81035 |
| P27797 | CALR | calreticulin | 811 |
| Q13492 | PICALM | phosphatidylinositol binding clathrin assembly protein | 8301 |
| Q969M2 | GJA10 | gap junction protein alpha 10 | 84694 |
| Q8IYJ3 | SYTL1 | synaptotagmin like 1 | 84958 |
| O43264 | ZW10 | zw10 kinetochore protein | 9183 |
| P35606 | COPB2 | coatomer protein complex subunit beta 2 | 9276 |
| Q96Q89 | KIF20B | kinesin family member 20B | 9585 |
| Q14677 | CLINT1 | clathrin interactor 1 | 9685 |
| O15027 | SEC16A | SEC16 homolog A, endoplasmic reticulum export factor | 9919 |
| Q8TBA6 | GOLGA5 | golgin A5 | 9950 |
