# Supplementary material for: Identification of calnexin as a diacylglycerol acyltransferase-2 interacting protein
Source: PLoS One. 2019 Jan 7;14(1):e0210396. doi: 10.1371/journal.pone.0210396 (PMC6322727; doi:10.1371/journal.pone.0210396)
Supplement: S5 Table — (DOCX) [file pone.0210396.s005.docx]

**Table S5. Overrepresentation of DGAT2-interacting proteins in KEGG pathway** **HAS-04141 (Protein processing in endoplasmic reticulum)**

| **C=166; O=21; E=4.71; R=4.46; PValue=6.75e-09; FDR=2.05e-06** |  |  |  |
| --- | --- | --- | --- |
|  |  |  |  |
| **UserID** | **Gene Symbol** | **Gene Name** | **Entrez Gene** |
| Q9HCU5 | PREB | prolactin regulatory element binding (Sec12) | 10113 |
| O95487 | SEC24B | SEC24 homolog B, COPII coat complex component | 10427 |
| Q92598 | HSPH1 | heat shock protein family H (Hsp110) member 1 | 10808 |
| Q9UGP8 | SEC63 | SEC63 homolog, protein translocation regulator | 11231 |
| P18850 | ATF6 | activating transcription factor 6 | 22926 |
| Q14697 | GANAB | glucosidase II alpha subunit | 23193 |
| P31689 | DNAJA1 | DnaJ heat shock protein family (Hsp40) member A1 | 3301 |
| P0DMV8 | HSPA1A | heat shock protein family A (Hsp70) member 1A | 3303 |
| P11021 | HSPA5 | heat shock protein family A (Hsp70) member 5 (Bip) | 3309 |
| P25685 | DNAJB1 | DnaJ heat shock protein family (Hsp40) member B1 | 3337 |
| P49257 | LMAN1 | lectin, mannose binding 1 (ERGIC53) | 3998 |
| Q8TAT6 | NPLOC4 | NPL4 homolog, ubiquitin recognition factor | 55666 |
| Q96IV0 | NGLY1 | N-glycanase 1 | 55768 |
| P14314 | PRKCSH | protein kinase C substrate 80K-H | 5589 |
| Q9P2E9 | RRBP1 | ribosome binding protein 1 | 6238 |
| Q99442 | SEC62 | SEC62 homolog, preprotein translocation factor | 7095 |
| P55072 | VCP | valosin containing protein (VCP) | 7415 |
| O76024 | WFS1 | wolframin ER transmembrane glycoprotein | 7466 |
| P27797 | CALR | calreticulin | 811 |
| P27824 | CANX | calnexin | 821 |
| Q9NZJ5 | EIF2AK3 | eukaryotic translation initiation factor 2 alpha kinase 3 (PERK) | 9451 |
